# Supplementary material for: Reconciling Mining with the Conservation of Cave Biodiversity: A Quantitative Baseline to Help Establish Conservation Priorities
Source: PLoS One. 2016 Dec 20;11(12):e0168348. doi: 10.1371/journal.pone.0168348 (PMC5173368; doi:10.1371/journal.pone.0168348)
Supplement: S1 Dataset — (ZIP) [file pone.0168348.s002.zip › Taxa/Serra Norte/SN_2013/N4E/Lista N4E-87.pdf]

# CAVIDADE N4E-0087

| Classe | Ordem | Fam/Outros | Gên/Outros | Espécie | S-U | U-S | Zon. |
|---|---|---|---|---|---|---|---|
| Arachnida | Acari | Prostigmata | | jovem | X | | |
| Arachnida | Acari | Rhagidiidae | | sp.1 | | X | P |
| Arachnida | Amblypygi | Charinidae | *Charinus* | sp.1 | | 1 | P |
| Arachnida | Amblypygi | Phrynidae | *Heterophrynus* | *longicornis* | 3 | | P |
| Arachnida | Araneae | Ctenidae | | jovem | 1 | | |
| Arachnida | Araneae | Nesticidae | *Nesticus* | sp.2 | X | | P |
| Arachnida | Araneae | Pholcidae | *Mesabolivar* | *aurantiacus* | X | | P |
| Arachnida | Araneae | Pholcidae | *Mesabolivar* | sp.1 | X | X | E/P |
| Arachnida | Araneae | Salticidae | | sp.4 | X | | E |
| Arachnida | Araneae | Theridiidae | *Episinus* | sp.1 | | X | P |
| Arachnida | Araneae | Theridiidae | *Theridion* | sp.1 | X | | E |
| Arachnida | Araneae | Theridiidae | *Theridion* | sp.4 | X | | E/P |
| Arachnida | Araneae | Theridiosomatidae | *Plato* | sp.1 | X | X | P |
| Arachnida | Opiliones | Cosmetidae | *Roquettea* | *singularis* | 1 | | E |
| Arachnida | Opiliones | Escadabiidae | | sp.2 | X | X | E/P |
| Arachnida | Opiliones | Stygnidae | *Protimesius* | *gracilis* | 2 | | P |
| Chilopoda | Scolopendromorpha | Scolopocryptopidae | *Tidops* | sp.1 | 2 | | E |
| Diplopoda | Polydesmida | Chelodesmidae | | jovem | 7 | | P |
| Diplopoda | Polydesmida | Cyrtodesmidae | | jovem | | X | E |
| Diplopoda | Polydesmida | Pyrgodesmidae | | jovem | | X | P |
| Entognatha | Collembola | Paronellidae | | sp.1 | X | | E |
| Entognatha | Collembola | Paronellidae | | sp.4 | | X | E/P |
| Insecta | Blattodea | | | jovem | | 3 | E/P |
| Insecta | Coleoptera | Curculionidae | Scolytinae | sp.4 | X | x | E |
| Insecta | Diptera | Culicidae | *Culex* | sp. | X | | E |
| Insecta | Diptera | Psychodidae | Phlebotominae | sp. | X | X | E/P |
| Insecta | Diptera | Tipulidae | Tipulinae | sp. | X | X | E/P |
| Insecta | Heteroptera | Cydnidae | | sp. | X | X | P |
| Insecta | Heteroptera | Reduviidae | Triatominae | sp. | | 1 | E |
| Insecta | Homoptera | Cixiidae | | jovem | X | X | E/P |
| Insecta | Hymenoptera | Braconidae | | sp.3 | X | | P |
| Insecta | Hymenoptera | Formicidae | *Camponotus* | sp.1 | X | X | E/P |
| Insecta | Hymenoptera | Formicidae | *Pachycondyla* | sp.2 | X | X | E |
| Insecta | Hymenoptera | Vespidae | | sp.3 | | X | E |
| Insecta | Isoptera | | | sp. | | X | P |
| Insecta | Lepidoptera | Noctuoidea | | sp.7 | 1 | 1 | E |
| Insecta | Orthoptera | Phalangopsidae | *Paraclodes* | jovem | 2 | 2 | E |
| Insecta | Orthoptera | Phalangopsidae | *Phalangopsis* | sp. | 5 | 9 | P |
| Insecta | Thysanura | Nicoletiidae | | sp.1 | X | | P |
| Gastropoda | Pulmonata | Bulimulidae | *Naesiotus* | sp. | | X | E |
| Gastropoda | Pulmonata | Subulinidae | | sp.3 | | X | E |
| Gastropoda | Pulmonata | Systrophiidae | *Happia* | sp. | X | X | P |
| Malacostraca | Isopoda | Philosciidae | | sp.1 | X | X | E/P |
| Symphyla | | Scutigerellidae | | sp. | | X | E |
| Amphibia | Anura | Strabomantidae | *Pristimantis* | cf. *fenestratus* | 10 | | E |
